# Exploring the CO_2_ Electrocatalysis Potential of 2D Metal–Organic Transition Metal–Hexahydroxytriquinoline Frameworks: A DFT Investigation

**DOI:** 10.3390/molecules29122896

**Published:** 2024-06-18

**Authors:** Yufeng Wen, Daguo Jiang, Zhangli Lai, Xianshi Zeng, Bo Liu, Yanan Xiao, Wen Ruan, Kai Xiong

**Affiliations:** 1 School of Mathematical Sciences and Physics, Jinggangshan University, Ji’an 343009, China; jgsuwyf@sina.com (Y.W.); jgsujdg1968@163.com (D.J.); liubo@jgsu.edu.cn (B.L.); yananxiao06@163.com (Y.X.); ruanwen@jgsu.edu.cn (W.R.); 2 Materials Genome Institute, National Center for International Research on Photoelectric and Energy Materials, School of Materials and Energy, Yunnan University, Kunming 650091, China; xiongkai@ynu.edu.cn; 3 Advanced Computing Center, Information Technology Center, Yunnan University, Kunming 650091, China

**Keywords:** electrocatalysis, metal–organic frameworks, TM–HHTQ, density functional theory

## Abstract

Metal–organic frameworks have demonstrated great capacity in catalytic CO_2_ reduction due to their versatile pore structures, diverse active sites, and functionalization capabilities. In this study, a novel electrocatalytic framework for CO_2_ reduction was designed and implemented using 2D coordination network-type transition metal–hexahydroxytricyclic quinazoline (TM–HHTQ) materials. Density functional theory calculations were carried out to examine the binding energies between the HHTQ substrate and 10 single TM atoms, ranging from Sc to Zn, which revealed a stable distribution of metal atoms on the HHTQ substrate. The majority of the catalysts exhibited high selectivity for CO_2_ reduction, except for the Mn–HHTQ catalysts, which only exhibited selectivity at pH values above 4.183. Specifically, Ti and Cr primarily produced HCOOH, with corresponding 0.606 V and 0.236 V overpotentials. Vanadium produced CH_4_ as the main product with an overpotential of 0.675 V, while Fe formed HCHO with an overpotential of 0.342 V. Therefore, V, Cr, Fe, and Ti exhibit promising potential as electrocatalysts for carbon dioxide reduction due to their favorable product selectivity and low overpotential. Cu mainly produces CH_3_OH as the primary product, with an overpotential of 0.96 V. Zn primarily produces CO with a relatively high overpotential of 1.046 V. In contrast, catalysts such as Sc, Mn, Ni, and Co, among others, produce multiple products simultaneously at the same rate-limiting step and potential threshold.

## 1. Introduction

Extensive fossil fuel use has led to significant environmental problems, particularly global warming, which is causing damage to ecosystems. This issue is primarily caused by the excessive discharge of CO_2_ [1]. The conversion of CO_2_ into valuable chemicals serves a dual purpose. Firstly, it can help alleviate environmental issues such as the greenhouse effect. Secondly, it can reduce reliance on conventional processes that heavily depend on fossil resources such as oil [2,3,4]. Consequently, there have been increasing efforts in recent years to process and utilize carbon dioxide [5]. Carbon dioxide reduction is a unique example where valuable hydrocarbon compounds, such as formic acid, methane, formaldehyde, ethanol, carbon monoxide, methanol, ethane, propylene, ethylene, etc., can be directly obtained through various avenues, including electrochemical [6,7], chemical reforming [8], photochemical [9], biochemical [10], and other pathways. Among these pathways, electrification-driven carbon dioxide conversion into useful chemicals is emerging as a strategy to accelerate the carbon cycle and mitigate environmental problems [11]. However, the small Gibbs free standard molar energy of CO_2_ (394.4 kJ mol^−1^) and its chemical passivation properties pose challenges. From a thermodynamic perspective, the conversion on CO_2_ into high-value-added chemicals or fuels (including ethane, methane, ethanol, and methanol) is a sluggish transition that typically includes several electron transfer steps [12]. Therefore, the development and synthesis of high-performance, low-cost catalysts for effective activation of inert CO_2_ molecules is a key scientific concern.

As a newly emerging subclass of 2D materials, 2D metal–organic frameworks (MOFs) are fabricated by self-assembly of metallic transition nodes and organic conjugated molecules that bear various orthogonal substituent moieties, such as -SeH, -SH, -OH, or -NH_2_ [13,14]. Two-dimensional MOFs possess diverse and exceptional structures, adjustable synthetic properties, outstanding porosity, and a high specific surface area, making them highly advantageous in various fields, including sensing, separation, and energy storage [15,16,17,18,19,20]. Due to the robust d–p conjugation within the 2D lattice and the compact p–p interactions perpendicular to the plane, 2D MOFs construct layered periodic frameworks that feature well-defined open channels, inherent porosity, superior charge carrier mobility, impressive conductivity, and adjustable catalytic and redox active sites [21,22,23,24,25,26]. Consequently, two-dimensional MOFs have made significant advancements in the realm of catalysis [27,28,29,30,31,32,33,34,35]. Furthermore, the strong affinity of MOFs for CO_2_ promotes the interaction of reactants and catalysts, facilitating effective progress of the reaction [36,37]. This unique advantage positions 2D MOFs as a popular and cutting-edge material in the field of CO_2_ electrocatalytic reduction.

In recent years, there has been considerable research into the electrocatalytic reduction of CO_2_ using MOFs. For instance, Zhu et al. [38]. developed an in situ method for the electrosynthesis of tubular Cu-MOFs, which were subsequently transformed into Cu dendrimer catalysts. The Cu-MOF was successfully fabricated in just 5 min, and the resulting Cu dendrimer catalyst exhibited efficient CO_2_ reduction to formate due to its rich surface area and abundance of active sites. This system achieved a remarkable 102.1 mA/cm^2^ current density and 98.2% selectivity in an ionic liquid electrolyte. Kang et al. [39]. utilized a substrate of copper foam and spatially resistive ligands to control the kinetics of MOF growth, leading to the rapid synthesis of numerous defective Cu-MOFs on the copper foam. In acetonitrile electrolyte ionic liquid, this catalyst demonstrated an efficient reduction of CO_2_ to formate, achieving 90.5% Faraday efficiency. Additionally, other researchers have demonstrated promising electrocatalytic performance in CO_2_ reduction through the incorporation of dopants into MOFs [40,41]. Despite the progress made in using conducting MOFs for CO_2_ reduction, there remain challenges with 2D MOF-mediated CO_2_ reduction reactions [42,43,44], primarily related to complicated pathways for electron transfer and energy barriers.

Chen et al. improved catalytic performance for CO_2_ by incorporating nitrogen-rich and electron-deficient tricyclic quinazoline (TQ) molecules into a 2D MOF structure [45]. They synthesized symmetric C_3_ 2,3,7,8,12,13-hexahydroxytricyclic quinazolines [46] and coordinated them with Ni^2+^ and Cu^2+^ to form square-planar M_3_(HHTQ)_2_ (M = Cu or Ni) 2D MOFs. These MOFs had a high metallic content, close to 20% w/w, with the metallic ions evenly distributed and firmly anchored in the lattice, indicating their potential for various applications. Cu_3_(HHTQ)_2_ exhibited exceptional electrochemical activity in CO_2_ to methanol, displaying high selectivity (53.6%), efficiency, and durability [47,48]. These experimental advancements motivated us to further investigate the potential applications of transition metal–HHTQ complexes in electrocatalytic CO_2_ reduction. Theoretical advances in recent years have had a significant impact on research in physics, chemistry, and materials science [49,50,51]. In this study, we prepared a series of TM–HHTQ metal–organic frameworks containing transition 3d metals and carried out a comprehensive investigation of their electrocatalytic CO_2_ reduction reaction (CO_2_RR) using density functional theory (DFT). Through computational simulations, we identified novel catalysts with potential reaction pathways and cost-effectiveness.

## 2. Results and Discussion

### 2.1. Characterization of the TM–HHTQ Structure

**Figure 1:**
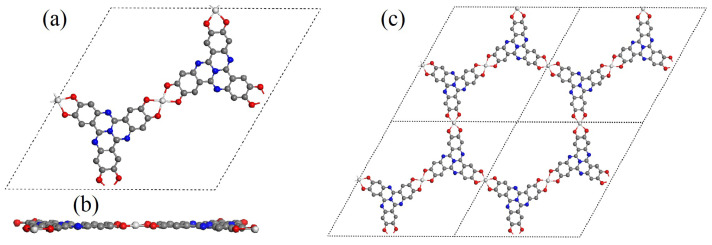
Single-cell structural view of TM–HHTQ. (**a**) displays the top view of a single cell, (**b**) displays the side view of a single cell, and (**c**) displays the top view of a 2 × 2 supercell.

Figure 1 illustrates the structures of transition metal–hexahydroxytricyclic quinazoline (TM–HHTQ). Panels (a) and (b) depict, respectively, a single-cell top and side view, while panel (c) displays the 2 × 2 supercell view. The top view shows that there are 42 C, 12 O, 8 N, and 3 transition metal atoms in each unit cell. In the hexahydroxytricyclic quinazoline molecule, each metallic atom coordinates with four oxygen atoms. All 10 transition metal atoms (from Sc to Zn) are located within the plane (Figure 1). The bond length between metallic and the nearest O ranges from 1.884 Å to 2.074 Å, as presented in Table 1. Notably, Sc-O bond exhibits the longest bond length of 2.074 Å due to the intrinsically larger atomic radius of Sc compared to the other 10 metals. Moreover, Hirshfeld charges were analyzed to investigate the electronic state of the monolayers. As indicated in Table 1, within the first group of transition metals, all metal atoms have a positive partial charge, while their nearest oxygen atoms exhibit a corresponding negative charge. This observation suggests that metal atoms undergo electron migration to the HHTQ monolayer, facilitating ionic bonding between metal and O atoms in addition to ligand bonding interactions. The metal atoms’ spin states have also been examined, and it was observed that Sc, Ni, and Zn exhibit non-spin states, while the remaining atoms possess spin states. Among these, Mn has the highest magnetic moment of 3.576 μB.

### 2.2. Stability of the TM–HHTQ Structure

Stability is a critical characteristic for evaluating the efficiency of the catalytic process. In order to evaluate TM–HHTQ monolayer stability, the binding energy of the TM–HHTQ monolayer and the cohesion energy of the bulk metal were calculated. The energy of cohesion was determined using the following relationship: $E_{c}=\left(E_{M(bulk)}-nE_{M}\right)/n$, where $E_{M(bulk)}$ represents the bulk energy, $E_{M}$ represents the single metal atom energy, and n represents the metal atom number within the bulk structure. The following expression was then used to determine the binding energy: $E_{b}=E_{TM-HHTQ}-E_{TM}-E_{HHTQ}$, where $E_{TM-HHTQ}$ represents the TM–HHTQ monolayer energy, $E_{TM}$ represents the single metal atom energy, and $E_{HHTQ}$ represents the hexahydroxytricyclic quinazoline monolayer energy.

**Figure 2:**
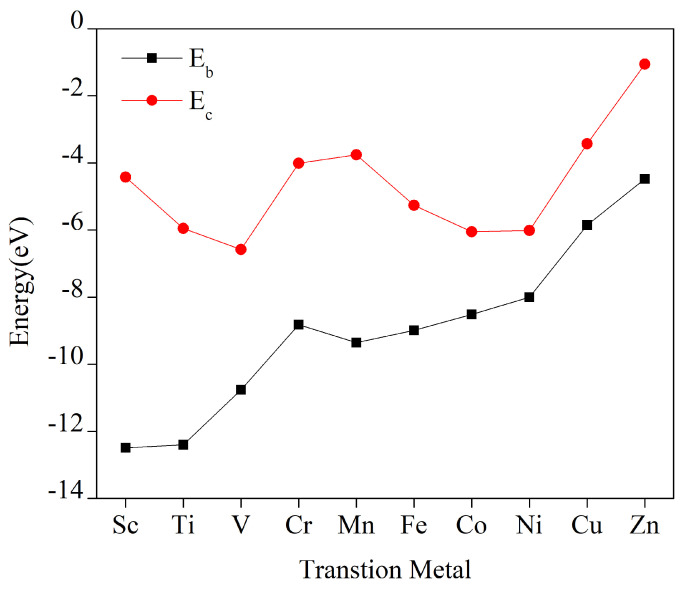
$E_{c}$ represents the bulk metal cohesion energy, $E_{b}$ represents the transition metal binding energy to the hexahydroxytricyclic quinazoline, and TM refers to the first 10 metals in the transition series.

In MOF catalysts, a stronger binding affinity between metal atoms and the substrate inhibits agglomeration of metal atoms, resulting in their uniform and stable incorporation into the substrate. Based on data from Table S1, the energy of cohesion of the bulk metal ranges from −6.577 eV to −1.055 eV, whereas the transition metal atom binding energy to the hexahydroxytricyclic quinazoline monolayer ranges from −14.494 eV to −4.475 eV. Cohesion energies of metal clusters, as shown in Figure 2, are consistently lower than the binding energies of the TM–HHTQ monolayers, demonstrating a preference for metal atoms to bind with hexahydroxytricyclic quinazoline rather than agglomerating with each other. Consequently, the TM–HHTQ monolayer exhibits excellent stability. Additionally, the interactions between these 10 metals (from scandium to zinc) and hexahydroxytricyclic quinazoline exhibit a gradual increase, suggesting a weakening strength of the binding bonds. This can be explained by the fact that oxygen is strongly non-metallic, whereas Sc to Zn are metals with gradually weakening metallicity. In general, stronger metallicity leads to stronger bonds with surrounding oxygen atoms, whereas weaker metallicity results in weaker bonds with oxygen atoms. Therefore, the gradually increasing trend of binding energies is reasonable.

### 2.3. Selectivity of TM–HHTQ for CO_2_RR and HER

The electrocatalytic CO_2_ reduction reaction commonly occurs in solution and involves multiple electronic steps. When an applied voltage is present, the reaction gradually involves proton and electron ($H^{+}+e^{-}$) pairs that are present in the solution. Upon CO_2_ molecule adsorption onto the surface of catalyst, the initial step of the protonation reaction produces two different intermediates, depending on where the hydrogen atom is added. Specifically, if a H atom is added to an O atom, a *COOH intermediate is formed. Conversely, when the H atom is added to a C atom, an *OCHO intermediate is formed. However, it is important to note that hydrogen can also attach to a catalyst’s metal atoms, causing a hydrogen evolution reaction (HER). This HER reaction competes with the CO_2_ reduction process and can affect the overall efficiency of the reaction. In essence, HER and the CO_2_ reduction reaction (CO_2_RR) are two competing reactions. Therefore, when designing CO_2_RR catalysts, it is crucial to consider the selectivity of the material for both CO_2_RR and HER.

**Figure 3:**
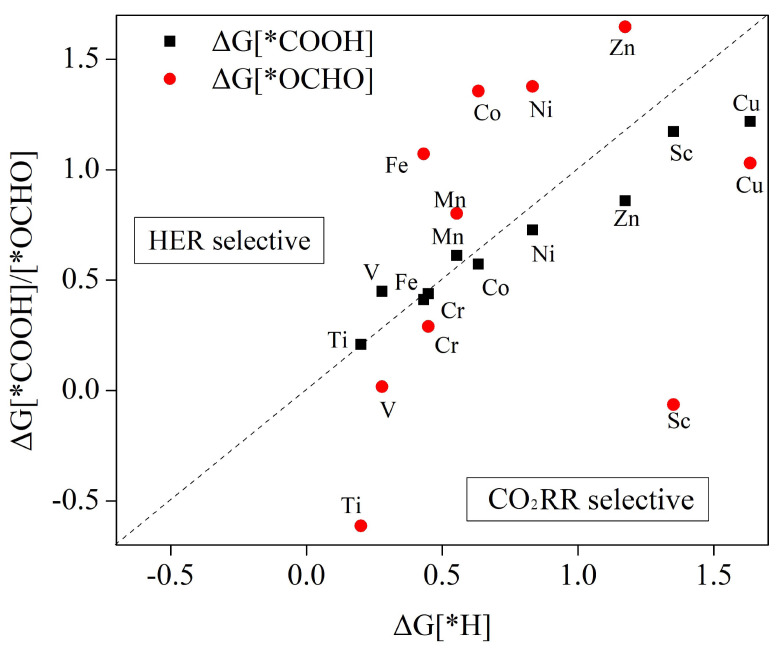
Changes in Gibbs free energy of CO_2_RR with HER protonated at step one of the surface of the TM–HHTQ monolayer. Below the dashed line are catalysts with high selectivity for CO_2_RR.

Figure 3 illustrates the changes associated with the Gibbs free energy for formation of *COOH, *OCHO, and *H by the protonation reaction in the first step. Specific values for these changes are provided in Table S2. From the analysis shown in Figure 3, it can be observed that the metals that include Fe, Mn, Co, Ni, and Zn have a higher tendency to form *COOH intermediates. On the other hand, elements such as Ti, V, Cr, Sc, and Cu tend to generate *OCHO, regardless of HER influence. When taking into account the competition from HER, it is evident that Sc, Cu, and Cr catalysts have lower Gibbs free energy compared to *H formation for both *OCHO and *COOH intermediates. This indicates that these catalysts effectively inhibit the HER reaction and possess strong electrocatalytic CO_2_ reduction activity. By inhibiting the HER, these catalysts demonstrate good selectivity for CO_2_ reduction, making them attractive for efficient CO_2_ conversion. For Ti and V, it was found that the Gibbs free energies were higher for the *COOH intermediate than for the *H intermediate, but smaller than those for *OCHO intermediate formation. The energies of formation for generation *OCHO intermediates are also higher than the metals Fe, Co, Ni, and Zn for generation of *H. However, Gibbs free energies for forming *COOH are lower than those for forming *H. Once active sites on the metal catalyst surface are filled with *OCHO or *COOH, there will not be any remaining active sites available to accept *H. This suggests that all six catalysts, Ti, Ni, Co, V, Zn, and Fe, are also catalytically active for the CO_2_RR. However, it can be observed from Figure 3 that the energy of formation is greater for Mn–HHTQ than for *H, irrespective of the formation of *OCHO or *COOH. This implies that as the site of activity accepts protons to produce *H; the main reaction that occurs is the precipitation of hydrogen.

**Figure 4:**
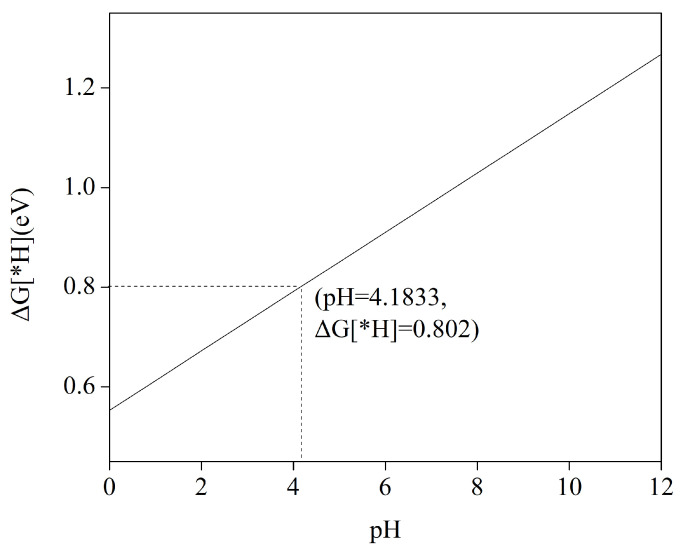
ΔG as a function of pH in the adsorption H-state of Mn–HHTQ.

When using Mn as a catalyst for CO_2_RR, adjusting the electrolyte pH is crucial for enhancing or inhibiting the hydrogen evolution reaction, which ultimately contributes to a smoother CO_2_RR process. This pH adjustment is particularly significant because the Gibbs free energy change for the generation of *H intermediates follows the relationship $\Delta G_{pH}=2.303k_{B}T\times pH$, in which $k_{B}$ represents Boltzmann’s constant, *T* represents reaction temperature (usually fixed at 298.15 K (room temperature)), and pH refers to electrolyte solution pH. When electrolyte solution pH is 0, Mn has a value of $\Delta G_{\left[*H\right]}$ of 0.553 eV. The value of $\Delta G_{\left[*H\right]}$ shows a linear relationship with pH. Figure 4 illustrates the correlation between Gibbs free energy ($\Delta G_{\left[*H\right]}$) and pH in the adsorbed hydrogen (H) status of the manganese (Mn) catalyst. The graph clearly shows a positive relationship, wherein, with increasing pH, the value of $\Delta G_{\left[*H\right]}$ increases progressively. For instance, the $\Delta G_{\left[*H\right]}$ for the Mn–HADQ complex was measured to be 0.802 eV when the pH of the solution reached 4.183. When the pH is greater than 4.183, there is a higher Gibbs free energy of *H intermediate formation compared to *COOH or formate *OCHO intermediate formation, indicating CO_2_RR selectivity.

### 2.4. Possible Product Pathways and Adsorption Energies

Due to the monoatomic nature of TM–HHTQ electrocatalytic carbon dioxide reduction, the production of multicarbon products is generally considered challenging. This limitation arises from the inability of the single-atom catalyst approach to achieve intermediate coupling that would enable the generation of C-C bonds. Theoretical predictions suggest that the monoatomic catalyzed CO_2_ reduction process primarily produces C_1_ products. The most commonly observed C_1_ products in the electrocatalytic reduction of CO_2_ include CH_3_OH, CH_4_, HCOOH, HCHO, and CO. Based on the electrocatalytic reduction scheme for obtaining C_1_ products from CO_2_ [52,53,54,55,56,57], the CO_2_ reduction to CO and HCOOH is a process with two electrons (2e). The pathways for converting carbon dioxide into CO and HCOOH are CO_2_ → *COOH → *CO → CO and CO_2_ → *OCHO → *HCOOH → HCOOH, respectively. HCHO formation from CO_2_ electrocatalytic reduction is a four-electron (4e) mechanism, with the reduction pathway being CO_2_ → *COOH → *CO → *CHO → *OCH_2_ → HCHO. Similarly, the generation of CH_3_OH in CO_2_ electrocatalytic reduction is a 6e mechanism, with the reduction pathway being CO_2_ → *COOH → *CO → *CHO → *OCH_2_ → *OCH_3_ → *OHCH_3_ → CH_3_OH. CH_4_ production from CO_2_ electrocatalysis is the most complicated process, and there are three potential pathways: (1) CO_2_ → *COOH → *CO → *COH → *C → *CH → *CH_2_ → *CH_3_ → * + CH_4_; (2) CO_2_ → *COOH → *CO → *CHO → *OCH_2_ → *OCH_3_ → O* + CH_4_ → *OH → H_2_O; (3) CO_2_ → *COOH → *CO → *CHO → *OCH_2_ → *OCH_3_ → *OHCH_3_ → *OH + CH_4_ → * + H_2_O. To predict each catalyst’s most likely product in the CO_2_ electrocatalytic reduction pathway, the first step is to calculate the catalyst’s energy of adsorption for C_1_ products.

**Figure 5:**
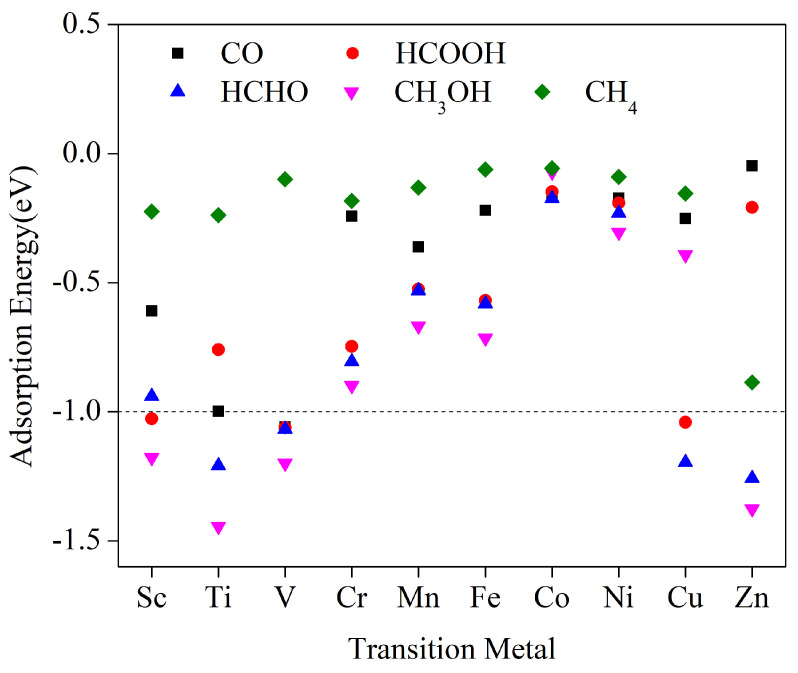
First-row transition metal (Sc-Zn) adsorption energies with hexahydroxytricyclic quinazoline (TM-HHTQ) on HCHO, HCOOH, CO, CH_3_OH, and CH_4_.

To ensure effective desorption and retrieval of electrocatalytic CO_2_ reduction products from the catalyst surface, it is crucial to prevent excessive adsorption of the products by the catalysts. This is essential to avoid catalyst poisoning and interruption of the catalytic process due to product desorption failure. To address this issue, we conducted calculations to determine these 10 TM–HADQ catalysts’ adsorption energies on all C_1_ products. The results of calculations, shown in Figure 5, indicate that all catalysts exhibit negative adsorption energies on the products. Lower values indicate stronger adsorption, making desorption of the products less likely. The specific values can be found in Table S3. Our findings show that Cr, Mn, Fe, Ni, and Co have relatively weak adsorption for all C_1_ products, as shown in Figure 5. Among them, Cr exhibits the highest adsorption energy for CH_3_OH, with a value of 0.899 eV. Therefore, based on their adsorption energies, these five catalysts have the potential to produce all C_1_ products. In the case of Sc–HHTQ, the adsorption energies for HCOOH and CH_3_OH were measured as 1.027 and 1.178 eV, respectively, indicating that these molecules have a low desorption probability and are more likely to be generated on the surface of Sc–HHTQ. Therefore, it is not necessary to prioritize HCOOH and CH_3_OH in the process of electrocatalytic CO_2_ reduction. However, since the catalyst’s adsorption on C_1_ products such as CO, CH_4_, and HCHO is weak, these products can be desorbed and generated more readily. Similarly, CO, HCHO, and CH_3_OH products do not need to be prioritized for Ti and V catalysts. Cu–HHTQ does not need to consider HCOOH and HCHO products, while the Zn catalyst does not need to consider HCHO and CH_3_OH products.

### 2.5. Reaction Pathways for Electrochemical Reduction of CO_2_

#### 2.5.1. With CO as the Main Product

We performed calculations to evaluate the free energy variation associated with each stage of protonation during the electrocatalytic CO_2_ reduction process. Our findings indicate that the Zn–HHTQ catalyst predominantly produces CO as a product.

**Figure 6:**
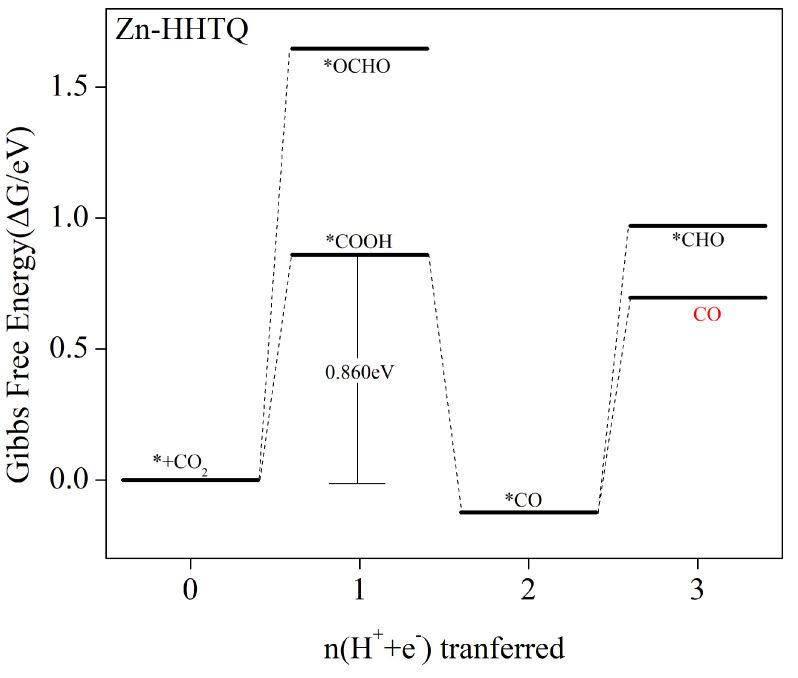
The Gibbs free energy profile of the CO_2_ reduction reaction (CO_2_RR) along the most favorable pathway for the Zn–HHTQ catalyst at potential zero is shown. CO_2_ adsorbed on the surface of the catalyst is considered to have zero free energy.

Based on the calculated changes in Gibbs free energy for each step of protonation in the electrocatalytic CO_2_ reduction process catalyzed by Zn–HHTQ (as depicted in Figure 6), it is evident that CO_2_ adsorbed on Zn–HHTQ undergoes the initial protonation reaction, resulting in *COOH or *OCHO intermediates under the influence of the external potential. However, our results indicate that, during the initial protonation step to form *OCHO, the Zn–HHTQ catalyst faces a higher energy barrier (as shown in Figure 3), leading to weaker reactivity compared to the competing HER. Therefore, we focus solely on the pathway that generates *COOH intermediates in the electrocatalytic CO_2_ reduction process. Table S4 shows the corresponding reaction equations and Gibbs free energy changes. Based on the results presented in Figure 6, it can be inferred that the *COOH intermediate formation is an energetically uphill process, involving a 0.860 eV energy barrier. Conversely, the subsequent 2e process that generates *CO intermediates is exothermic, with diminishing free energy, and, therefore, occurs readily. After the generation of the *CO intermediate, additional protonation reactions may take place, resulting in the production of *CHO or *COH, or CO desorption may occur to produce the final product. It should be noted that the further protonation step is an endothermic free energy increase process, requiring overcoming a 1.095 eV or 1.802 eV energy barrier. In contrast, the barrier energy required for the CO desorption step is relatively low, at 0.819 eV. Consequently, the step in which CO desorption occurs and products are produced is more favorable. In summary, the overall catalytic route can be described by *+ CO_2_→ *COOH → *CO → CO. The rate-determining step is *+ CO_2_ + H^+^ + e^−^ → *COOH, corresponding to a 0.860 V limiting potential.

#### 2.5.2. The Principal Product of Catalysis Is HCOOH

**Figure 7:**
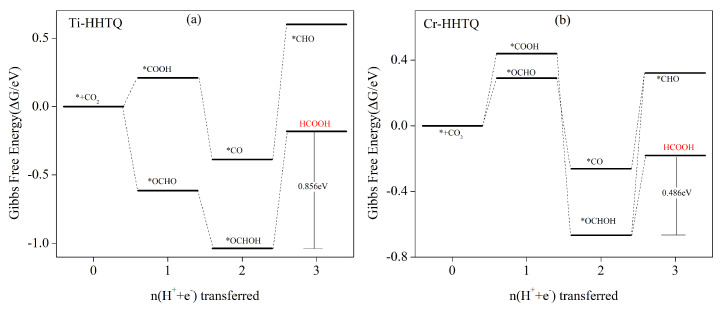
Plots of Gibbs free energy for (**a**) Ti–HHTQ and (**b**) Cr–HHTQ, shown with zero potential, depicting the most favorable pathway for the CO_2_ reduction reaction (CO_2_RR). The zero point of the free energy scale is defined as the energy of CO_2_ in the gas phase relative to a clean catalyst surface.

The calculations reveal that the primary product of the CO_2_ reduction reaction using both Ti–HHTQ and Cr–HHTQ catalysts is HCOOH. Figure 7a shows the step diagrams of Gibbs free energy for each step of protonation in Ti–HHTQ catalysts, while Table S5 provides the corresponding chemical equations and Gibbs data. Similarly, Figure 7b illustrates the stepwise Gibbs free energy diagrams for each step of protonation in Cr–HHTQ catalysts, and Table S6 lists the corresponding chemical equations and Gibbs data. Figure 5 indicates that the adsorption energy of Ti–HHTQ is relatively low for CH_4_ and HCOOH, but strong for other C_1_ products, rendering these products unable to be generated through desorption from the Ti–HHTQ surface. Therefore, we only focus on the generation of CH_4_ and HCOOH when analyzing the CO_2_ reduction pathway for the Ti–HHTQ catalyst. When CO_2_ is adsorbed onto the Ti–HHTQ catalyst surface, the initial protonation reaction occurs under an external voltage to produce *COOH. *OCHO formation is an exothermic process, leading to a free energy decrease. On the other hand, *OCHOH formation also occurs exothermically, leading to a free energy decrease. In the subsequent second step of the protonation reaction, both the generation of *CO and *OCHOH intermediates are characterized by a free energy decrease. There are several possibilities for the third step of the protonation reaction when combining pathways to produce both CH_4_ and HCOOH. (1) *CO can undergo a transformation to a *CHO/*COH reaction, which is characterized by an absorption of heat and an increase in free energy, with a corresponding change in Gibbs free energy of 0.986 eV/2.007 eV (Table S5); (2) *OCHOH can undergo a transformation to a *CHO/*OCH reaction, which is also characterized by an absorption of heat and an increase in free energy, corresponding to a change in Gibbs free energy of 1.637 eV/2.092 eV (Table S5); (3) HCOOH can desorb to produce the final product, which is characterized by an absorption of heat and an increase in free energy. This pathway involves a 0.856 eV energy barrier and is considered the most probable pathway for the 3e process. Therefore, the most likely product generated by the Ti-HHTQ catalyst for the electrocatalytic CO_2_ reaction is HCOOH, with a pathway of * + CO_2_ → *OCHO → *OCHOH → HCOOH. The rate-controlling step for the entire process is *OCHOH → * + HCOOH, corresponding to 0.856 V. The reaction pathway of HCOOH is *OCHO → *OCHO → *OCHOH → *OCHO → *OCHO → *OCHO → *OCHO → *OCHOH.

Utilizing Cr–HHTQ as a catalyst for the electrocatalytic reduction of CO_2_, the first step of the protonation reaction to produce *OCHO exhibits 0.290 eV—the lowest barrier (Table S6). The subsequent 2e process, which yields either *OCHOH or *CO intermediates, is characterized by exothermal free energy-reduced reactions and can proceed spontaneously. However, in the 3e process that follows, all of the reactions exhibit endothermic characteristics with an increase in free energy. Among these reactions, the HCOOH desorption process requires the lowest 0.486 eV energy barrier. In summary, the primary product of Cr–HHTQ in CO_2_ electrocatalytic reductive processes is HCOOH, with the pathway involving the successive generation of *OCHO, *OCHOH, and HCOOH. The reaction rate-determining step is the conversion of *OCHOH to * and HCOOH, corresponding to a limiting potential of 0.486 V.

#### 2.5.3. The Major Catalytic Product Is CH_4_

**Figure 8:**
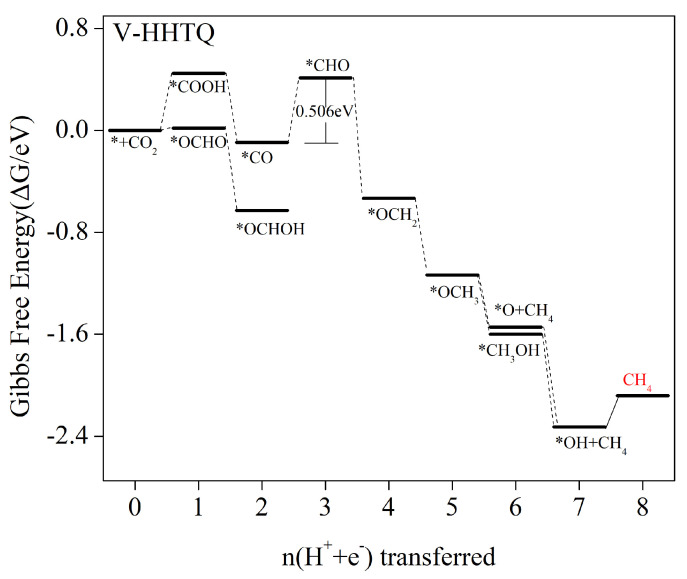
Gibbs free energy profile of CO_2_RR at zero potential following the most favorable V–HHTQ pathway. The CO_2_ molecules adsorbed on the surface of the catalyst have free energy of zero.

Based on Figure 5, it is evident that V–HHTQ exhibits strong adsorption to the majority of C_1_ products, resulting in CH_4_ production only. Therefore, we focus only on the pathway leading to the CH_4_ product. Figure 8 illustrates the energy barrier diagrams of every step in the V–HHTQ electrocatalytic CO_2_ reduction process. The specific values of Gibbs free energy changes during the different steps of protonation are available in Table S7. After CO_2_ adsorption onto the V–HHTQ surface, initial protonation to form *COOH/*OCHO involves an increase in energy, requiring an energy barrier of 0.449 eV/0.018 eV to be overcome. An exothermic reaction and energy loss occur in the subsequent 2e process to form *CO/*OCHOH. In the subsequent 3e process, *CO can convert to *CHO/*COH or *OCHOH can convert to *CHO/*OCH. Based on the information provided in Table S7, all potential 3e processes are characterized as heat-absorbing reactions with an increase in free energy. Among these reactions, *CO→*CHO has the lowest barrier energy to overcome, making it likely to be the most dominant pathway. After *CHO intermediate formation, subsequent steps of protonation are typically free energy-decreasing exothermic reactions that can easily occur until the final CH_4_ product is formed. Thus, the primary CO_2_ reduction product of V–HHTQ electrocatalysis is CH_4_. The reaction path is as follows: * + CO_2_ -> *COOH -> *CO -> *CHO -> *OCH_2_ -> OCH_3_ -> *O + CH_4_/*CH_3_OH -> *OH + CH_4_ -> CH_4_ + H_2_O. The reaction that determines the rate of the whole process is *CO + H_2_O + H^+^ + e^−^ -> *CHO + H_2_O, corresponding to a 0.506 V limiting potential.

#### 2.5.4. Main Product of Catalysis Is CH_3_OH

**Figure 9:**
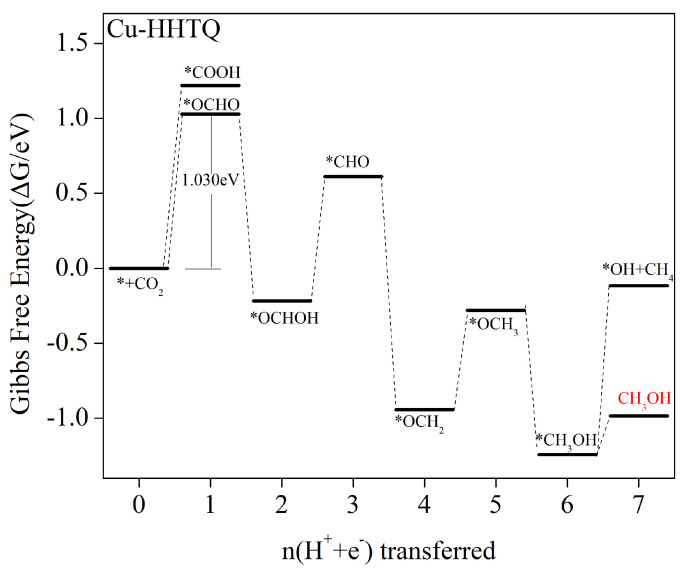
Profile of the Gibbs free energy for CO_2_RR at zero potential along the most favorable Cu–HHTQ pathway. The CO_2_ molecules adsorbed on the catalyst surface have a free energy of zero.

The energy diagrams depicting the protonation steps in Cu–HHTQ electrocatalytic CO_2_ reduction are presented in Figure 9. The corresponding Gibbs free energy change equations and values for the individual steps can be found in Table S8. When Cu–HHTQ is employed as an electrocatalyst for CO_2_ reduction, the initial protonation step undergoes an endothermic reaction with an increase in free energy. The *COOH intermediate formation results in a change in Gibbs free energy of 1.219 eV, whereas *OCHO intermediate generation results in a 1.030 eV Gibbs free energy change. Thus, the formation of the *OCHO intermediate is overwhelmingly dominant. Following the 2e process, *OCHOH formation occurs exothermically as a free energy-reducing reaction. The energy barriers necessary for the 3e conversion are 0.829 eV and 1.255 eV for *OCHOH to *CHO and *OCH, respectively. Hence, this process produces *CHO intermediates with complete dominance. In the subsequent 4e process, the formation of *OCH_2_ intermediate occurs as a low-free-energy exothermic reaction. However, subsequent 5e processing requires surpassing a 0.662 eV energy barrier to achieve protonation and to generate *OCH_3_ intermediates. The formation of *CH_3_OH in the subsequent 6e process is exothermic with a free energy decrease. However, there is a 1.125 eV energy barrier to overcome for further protonation after the generation of *CH_3_OH. In contrast, the barrier to desorption of CH_3_OH and production of the final product is only 0.257 eV. Thus, the generation of the CH_3_OH product marks the end of the reaction. Overall, the Cu–HHTQ electrocatalytic CO_2_ reduction process produces CH_3_OH products simultaneously. The path is as follows: * + CO_2_ → *OCHO → *OCHOH → *CHO → *OCH_2_ → *OCH_3_ → *CH_3_OH. In this process, the rate-determining step is * + CO_2_ + H^+^ + e^−^ → *OCHO, which has a limiting potential of 1.030 V.

#### 2.5.5. Simultaneous Generation of Two Products: HCHO and CH_3_OH

**Figure 10:**
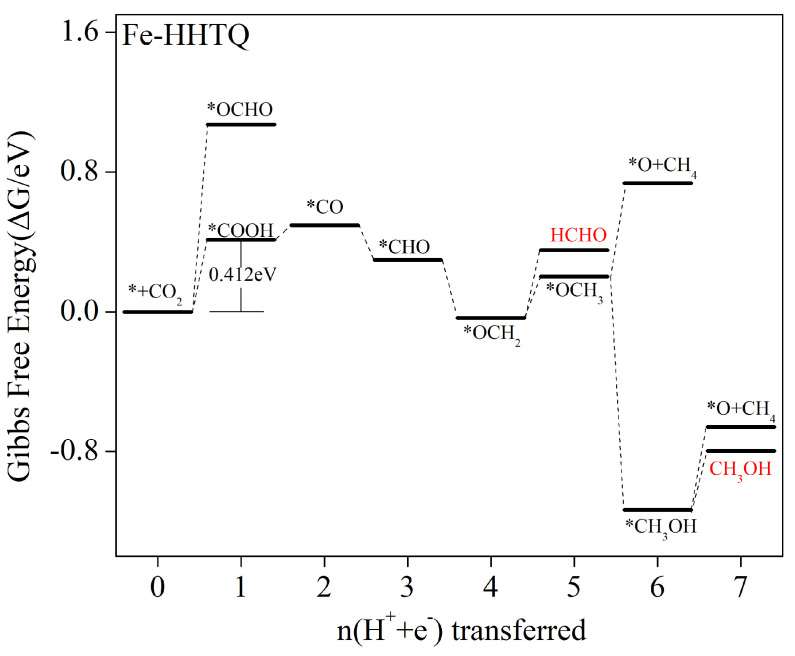
The Fe–HHTQ at zero potential Gibbs free energy profile is shown alongside the CO_2_RR most favorable path. Zero free energy is defined as the energy of the CO_2_ in the gas phase compared to a pure surface of the catalyst.

Figure 10 presents the Gibbs free energy changes for each protonation step in the Fe–HHTQ electrocatalytic reduction of CO_2_ process. The accompanying equations and values of change in Gibbs free energy are presented in Table S9. During the electrocatalytic CO_2_ reduction process using Fe–HTQ, the initial protonation step involves heat absorption and has an increasing free energy. The Gibbs free energy changes are 0.412 eV and 1.072 eV for *COOH and *OCHO intermediate formation, respectively. The dominant formation is, therefore, that of the *COOH intermediates. In the subsequent 2e process, a lower energy barrier (0.084 eV) must be overcome. The subsequent 3e and 4e processes are reduced-free-energy exothermic reactions, allowing the automatic occurrence and generation of *OCH_2_ intermediates. After the generation of the *OCH_2_ intermediate, HCHO desorption may take place, resulting in the generation of products. The energy barrier to be overcome is 0.338 eV. Alternatively, additional protonation reactions can occur, resulting in the generation of *OCH_3_ intermediates. The subsequent 6e process is a free-energy-reduced exothermic reaction, yielding the *CH_3_OH intermediate. After the generation of *CH_3_OH intermediates, the subsequent *O intermediate protonation must overcome a relatively small 0.475 eV barrier energy. Then again, CH_3_OH desorption and generation of product only require an energy barrier of 0.337 eV to overcome. Thus, in this step, CH_3_OH desorption occurs to generate the product and the reaction is terminated. Overall, the Fe–HTQ electrocatalytic CO_2_ reduction produces both HCHO and CH_3_OH products. The pathway is described as follows: * + CO_2_ → *COOH → *CO → *CHO → *OCH_2_ → HCHO/*OCH_3_ → *CH_3_OH → CH_3_OH. The rate-determining step is * + CO_2_ + H^+^ + e^−^ → *COOH, having a 0.412 V limiting potential.

#### 2.5.6. Formation of CO, HCHO, CH_3_OH, and CH_4_ as Products

**Figure 11:**
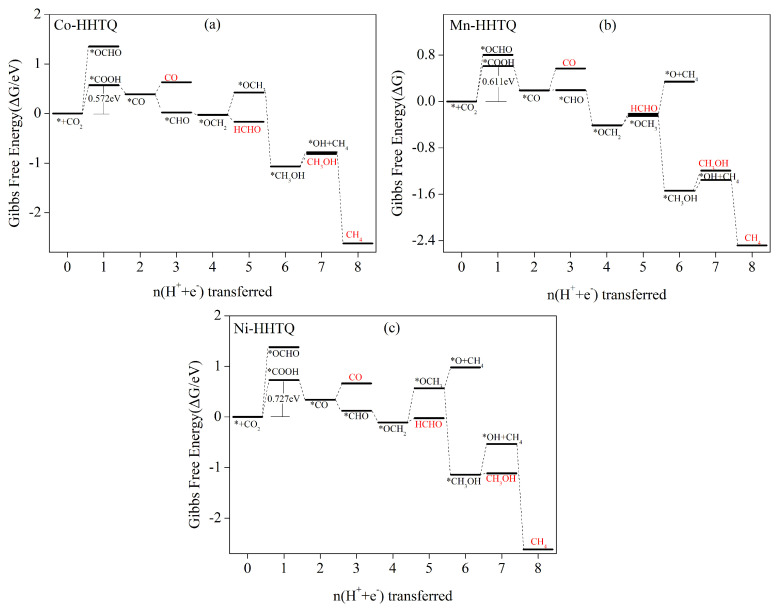
Zero–potential Gibbs free energy curves of (**a**) Co–HHTQ, (**b**) Mn–HHTQ, and (**c**) Ni–HHTQ following the optimal path of CO_2_RR. The zero point of free energy is defined as a CO_2_ molecule in the gas phase on the surface of a clean catalyst.

Computational studies have revealed that the Co–HHTQ, Mn–HHTQ, and Ni–HHTQ catalysts exhibit low selectivity in the electrocatalytic CO_2_ reduction process, producing four products simultaneously, namely, CO, HCHO, CH_3_OH, and CH_4_. Figure 11a–c illustrates stepwise Gibbs free energy diagrams of the intermediates in electrocatalytic pathway for the three catalysts, Co–HHTQ, Mn–HHTQ, and Ni–HHTQ, respectively. Detailed electrocatalytic step reaction equations and corresponding Gibbs free energies can be found in Tables S10–S12. In the Co–HHTQ process for electrocatalytic CO_2_ reduction, the initial step is a heat-absorbing protonation reaction with increasing free energy. Compared to the formation of *OCHO intermediates, the energy barrier required to generate *COOH intermediates is lower, measuring 0.572 eV (as shown in Table S10), providing an advantage. Therefore, our focus is on the reaction pathway that yields *COOH. Subsequently, a 2e process generates *CO as a reduced-free-energy exothermic reaction. Following *CO formation, protonation to *COH requires overcoming a 1.378 eV energy barrier (as shown in Table S10). Hence, this pathway is not considered. However, the generation of *CHO is a reaction that is exothermic and has reduced free energy. Simultaneously, CO desorption has to overcome a lower energy barrier (0.241 eV) compared to 0.572 eV for *COOH formation. Consequently, after the *CO intermediate is formed, the process of protonation towards the generation of *CHO can occur alongside CO desorption and further protonation steps. Similarly, the desorption and protonation of HCHO and CH_3_OH as products in the subsequent electrocatalytic steps can also occur simultaneously until the CH_4_ product is generated at the end of the 8e process. In summary, Co–HHTQ as a CO_2_-reduction electrocatalyst leads to the simultaneous production of CO, HCHO, and CH_3_OH as products. The pathway can be summarized as such: * + CO_2_ → *COOH → CO/*CHO → OCH_2_ → HCHO/OCH_3_ → *CH_3_OH → CH_3_OH/*OH + CH_4_ → CH_4_. The rate-determining step is * + CO_2_ + H^+^ + e^−^ → *COOH, with a limiting potential of 0.572 V.

The electrocatalytic CO_2_-reduction processes of Mn–HHTQ and Ni–HHTQ exhibit similar behavior to that of Co–HHTQ, as they also generate CO, HCHO, and CH_3_OH products simultaneously. The reaction pathways of Mn–HHTQ and Ni–HHTQ for CO_2_ electrocatalytic reduction are analogous to those of Co–HHTQ: * + CO_2_ → *COOH → CO/*CHO → OCH_2_ → HCHO/OCH_3_ → *CH_3_OH → CH_3_OH/*OH + CH_4_ → CH_4_. The rate-limiting step for both Mn–HHTQ and Ni–HHTQ is * + CO_2_ + H^+^ + e^−^ → *COOH, with 0.611 V and 0.727 V limit potentials.

#### 2.5.7. Three Products, CO, HCHO, and CH_4_, Are Generated Simultaneously

**Figure 12:**
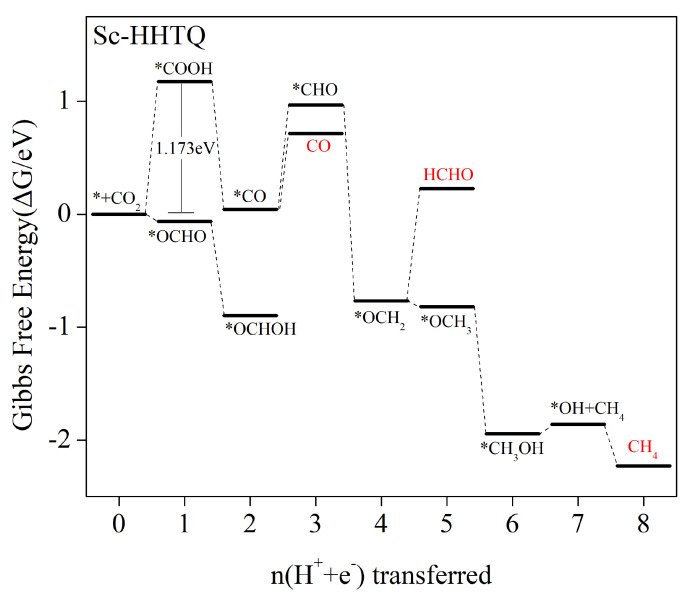
Zero-potential Gibbs free energy profile of CO_2_RR along the most favorable Sc–HTQ pathway. The CO_2_ molecules adsorbed on the catalyst surface have zero free energy.

Based on Figure 5, it is evident that the adsorption of HCOOH and CH_3_OH during Sc–HTQ electrocatalytic CO_2_-reduction is excessively strong, leading to inhibition of the catalytic process and hindering the production of the desired product. As a result, the generation pathways of HCOOH and CH_3_OH are not considered in the Sc–HTQ electrocatalytic CO_2_ reduction process. The free-energy steps per protonation step in the Sc–HTQ electrocatalytic CO_2_ reaction are illustrated in Figure 12, while the chemical reaction equations with corresponding data for each step’s Gibbs free energy are presented in Table S13. In first protonation step, *OCHO formation is an exothermic process, leading to a decrease in free energy. Similarly, the subsequent generation of *OCHOH proceeds exothermically at reduced energy. However, after the formation of *OCHOH intermediates, the energy barriers for the next protonation step to produce *CHO and *OCH are both very high, with values of 1.865 eV and 2.031 eV, respectively. Therefore, this pathway is not considered. On the other hand, only the step of *COOH generation in the reaction pathway to produce the *COOH intermediate presents the highest energy barrier to overcome, which is 1.173 eV, until the 8-electron process generates the product CH_4_. Therefore, in the Sc–HTQ electrocatalytic reduction of CO_2_, the *COOH formation step represents the only step with the highest energy barrier to overcome throughout the entire reaction. In summary, the Sc–HTQ electrocatalytic CO_2_ reduction process results in the simultaneous generation of CO, HCHO, and CH_4_ products, with the following pathway: * + CO_2_ → *COOH → *CO → CO/*CHO → OCH_2_ → HCHO/OCH_3_ → *CH_3_OH → *OH + CH**_4_** → CH_4_. The pathway step that determines the rate is the * + CO_2_ + H^+^ + e^−^ → *COOH step, with 1.173 V limiting potential.

### 2.6. Electronic Structure Analysis

Based on the analysis of the change in Gibbs free energy in every stage, the rate-determining step, the limiting potential, and the corresponding main product for each catalyst are discussed in Section 2.5. Table 2 presents the rate-determining steps, limiting potentials, and overpotentials for 10 metal-catalyzed processes. By observing Table 2, it is evident that six catalysts, namely, Cr, V, Ti, Fe, Cu, and Zn, exhibit favorable product selectivity. On the other hand, the remaining four catalysts generate multiple products simultaneously under the same limiting potential. The overpotentials of Sc, Cu, and Zn catalysts are comparatively higher and fall within the range of 0.9–1.3V, while the other catalysts have overpotentials in a range of 0.236–0.78 V, which compare favorably with Cu (211) (η = 0.77 V) and Pt (111) (η = 0.46 V), the most active step surfaces [58]. In a previous study, the electrocatalytic CO_2_-reduction properties of TM–THQ, a composite of 3d transition metals with tetrahydroxybenzoquinone, were theoretically evaluated. The findings revealed that the limiting and overpotentials of Ti–THQ were the highest, measuring 1.043 V and 1.212 V, respectively, while the other monolayered catalysts exhibited relatively lower potentials, within the range of 0.172 V to 0.952 V [57]. And the limiting potential of TM–HITP constructed from 10 3d transition metals and hexaiminotriphenylene for electrocatalytic CO_2_ reduction is between 0.296–0.738V [54]. These results suggest that TM–HHTQ holds promise for electrocatalytic CO_2_ reduction comparable to those of TM–THQ and TM–HITP. In summary, the catalysts Ti, V, Cr, and Fe demonstrate promising potential as MOF catalysts for electrocatalytic CO_2_ reduction due to their favorable attributes of low overpotentials and good product selectivity.

**Figure 13:**
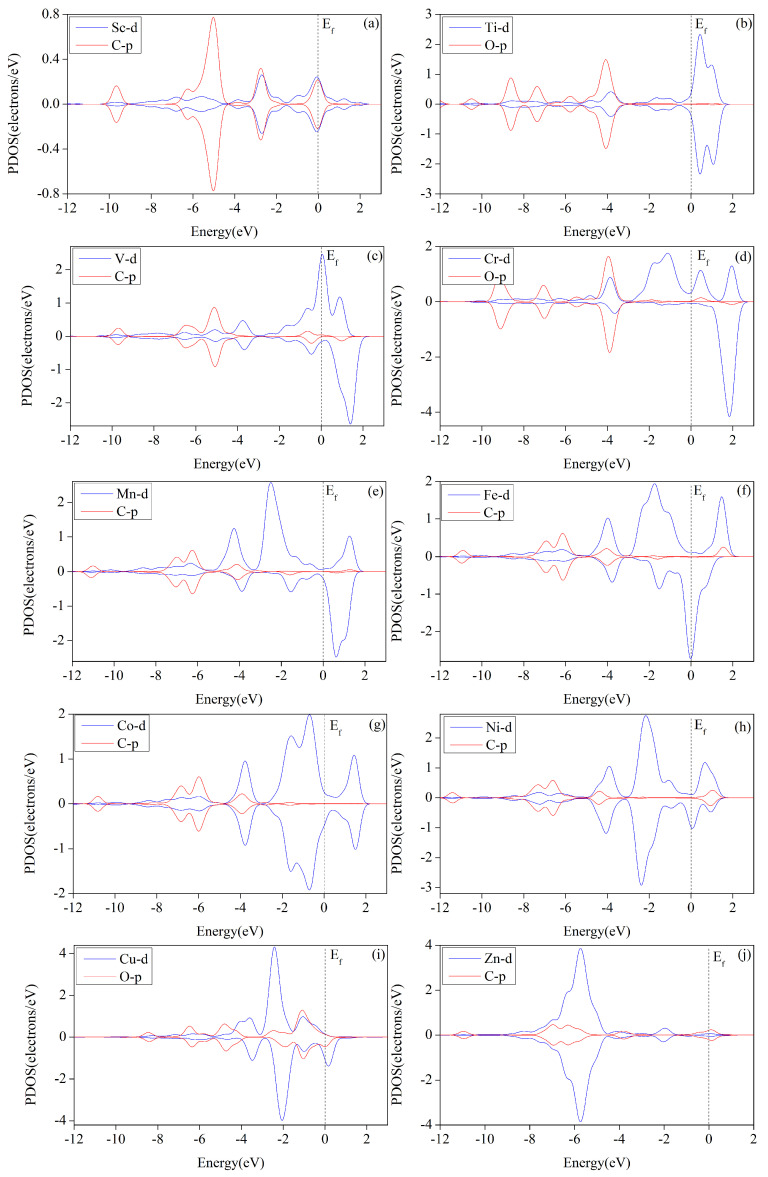
The predicted density of partial states shows the adsorption of *COOH on Sc (**a**), Fe (**f**), Mn (**e**), Ni (**h**), Co (**g**), and Zn (**j**), in addition to *OCHOH adsorbed upon Cr (**d**) and Ti (**b**), *CHO adsorbed upon V (**c**), and *OCHO adsorbed upon Cu (**i**).

The theory of metal-ligand bonding in metal–organic catalysts proposes that the interaction of catalyst and intermediate occurs primarily through σ- and π-bonds. Different ligands and intermediates form distinct σ-bonds and π-bonds to modulate the activity of metal catalysts, thereby influencing the selectivity of products. In the rate-determining step, the chemical bonding between the catalyst and the intermediate plays a decisive role. Stronger bonding between the catalyst and the ligand in the rate-determining step leads to a more stable intermediate, requiring a higher external voltage to promote the reaction, thus resulting in a higher limiting potential for the catalyst. Conversely, this corresponds to a lower limiting potential. Therefore, we calculated the partial density of states of intermediates in the rate-determining steps for each catalyst. Figure 13 demonstrates the evident overlapping of the metal atoms’ 3d orbitals and the O or C atoms’ 2p orbitals in intermediate species, determining the reaction step (*OCHOH, *OCHO, *CHO, or *COOH). The interaction strength between the TM–HHTQ monolayer and intermediate is evident in both spin-up and spin-down states. These TM–HHTQ frameworks exhibit a density of electronic states other than zero in the Fermi energy level, indicative of metallization, with V and Fe showing the most pronounced effects. The overlap of the 3d and 2p orbitals in Figure 13a,i is more pronounced than in Figure 13d,f, suggesting that Sc and Cu have stronger interactions with the corresponding intermediates than Fe and Cr. The stability of the adsorbed intermediate system increases with stronger interaction, leading to a higher energy barrier that must be overcome for a catalytic reaction to occur. This ultimately results in a greater free energy increase in the critical step in the catalytic reduction of carbon dioxide by Sc–HTQ and Cu–HTQ, leading to a more negatively biased reaction limiting potential. The obtained limiting potentials $U_{L}$ for catalytic reduction of CO_2_ by Sc–HTQ and Cu–HTQ, as shown in Table 2, are 1.173 eV and 1.030 eV, respectively. These values are greater than those observed for Cr and Fe catalysts. These observations are in agreement with the results obtained from the PDOS (Projected Density of States) analysis.

## 3. Calculation Details

We conducted all calculations using the Dmol3 software package, a program that uses spin-polarized DFT [59]. We took into account correlations between electrons with the Perdew–Burke–Ernzerhof (PBE) functional [60], which is a specific generalized gradient approximation (GGA) form. We used the DNP basis set and implemented the DSPP approximation, incorporating relativistic corrections and utilizing a single effective potential to represent the kernel electrons [61]. To enhance the characterization of molecule adsorption on the surface and capture the influence of low long-range interaction forces among layers, we incorporated a van der Waals-type corrective (DFT-D2) [62,63,64,65] into our calculations. To improve agreement with experimental data, we implemented the conductor approximate shielding model (COSMO) as a solvation method in our study. We chose water, with a relative dielectric constant of ϵ=78.54, for simulating the solvent effect on all systems [66]. To prevent interactions between neighboring heterogeneous nodes, we selected a 25 Å thickness of the vacuum layer. We used a 10^−6^ eV energy convergence criterion to improve accuracy in our calculations. For structure optimization, we employed a Monkhorst Pack K-point grid of 3 × 3 × 1, while, for electronic structure calculations, we used a Monkhorst Pack K-point grid of 6 × 6 × 1.

Equation (1) is used to define energy of adsorption (*E_ads_*), which provides a gauge of interaction strength between the HHTQ monolayer and intermediate.

(1) $$E_{ads}=E_{substrate-adsorbate}-E_{substrate}-E_{adsorbate}$$

In this equation, the term $E_{substrate-adsorbate}$ represents the total energy of the small molecules that have been adsorbed onto the surface of TM–HHTQ. The term $E_{substrate}$ refers to the energy of the TM–HHTQ substrate, while the term $E_{adsorbate}$ refers to the energy of the individual small molecules that have been adsorbed. Since CO_2_RR involves various reaction pathways, we have incorporated the concept of Gibbs free energy to identify the most favorable reaction pathways. Gibbs free energy is a thermodynamic parameter that takes into account both the enthalpy and entropy changes in a chemical reaction, and it is used to predict whether a reaction will occur spontaneously or not. By calculating the Gibbs free energy for different reaction pathways, we can identify the most favorable pathway for CO_2_RR on the TM–HHTQ substrate.

For reactions involving transfer of electrons, energy can be determined using a standard hydrogen electrode model, as presented by Nørskov et al. [52,54]. Equation (2) outlines the calculation of Gibbs free energy.

(2) $$\Delta G=\Delta E+\Delta E_{ZPE}-T\Delta S+\Delta G_{PH}+\Delta G_{U}$$

where the reaction energy is ΔE, the zero-point energy and entropy changes are $\Delta E_{ZPE}$ and ΔS (using experimental values for the energy of zero point and entropy of small molecules), and T is 298.15 K for the reaction’s thermodynamic temperature. $\Delta G_{pH}$ is the adjustment to free energy caused by the variation in the acidity of the solution (varying concentrations of $H^{+}$ ions) ($\Delta G_{pH}=2.303k_{B}TpH$), which is considered to be 0 in solutions containing acids. $\Delta G_{U}$ is the free energy correction due to difference in electrode potentials, which can be obtained using Equation (3):

(3) $$\Delta G_{U}=-neU$$

where *n* represents electron transfer and *U* is the applied electrode potential. Ultimate potential ($U_{L}$) and overpotential (η) are critical parameters in assessing the effectiveness of activity of the catalyst. The ultimate potential can be calculated from Equation (4):

(4) $$U_{L}=-\Delta G_{max/ne}$$

$\Delta G_{max}$ refers to free energy change during the step that determines the rate. Overpotential is determined by subtraction of equilibrium potential (Uequilibrium) from limiting potential, as given in Equation (5):

(5) $$\eta =U_{equilibrium}-U_{L}$$

## 4. Conclusions

In this study, we studied carbon dioxide electrocatalytic reduction using density functional theory calculations on a 2D coordination material composed by TM–HHTQ. Our results indicate that the energy levels of HHTQ ligand-bound metal atoms in the 10 TM–HHTQ monolayers are high enough to allow for stable dispersal of the HHTQ substrate. Most of the catalysts demonstrated favorable selectivity for CO_2_RR, although Mn–HHTQ displayed selectivity for CO_2_RR only at pH levels above 4.183. The primary product of Ti and Cr catalysts was found to be HCOOH, corresponding to overpotentials at 0.606 V and 0.236 V, respectively. V catalyst produced CH_4_ as the main product, with an overpotential of 0.675 V. Fe catalyst generated HCHO as the main product, with an overpotential of 0.342 V. Based on these findings, Ti, Cr, V, and Fe are promising CO_2_RR electrocatalysts with desirable product selectivity and low overpotentials. The Cu catalyst predominantly produces CH_3_OH, with 0.96V overpotential. The Zn catalyst primarily yields CO, with a relatively high overpotential of 1.046 V. On the other hand, Sc, Mn, Ni, and Co catalysts display simultaneous generation of multiple products at the same rate-determining step and limiting potential.

## Figures and Tables

**Table 1:** Several structural characteristics of the TM–HHTQ monolayer were evaluated, including analysis of the Hirshfeld charge of the metal atom ($Q_{TM}$) and its closest oxygen atom ($Q_{O}$), as well as an analysis of the spin state of the metal atom and bond width to the closest oxygen atom ($R_{TM-O}$).

TM-THQ	$Q_{\mathrm{TM}}$/e	Spin-TM	$Q_{O}$/e	$R_{\mathrm{TM}-O}$/Å
Sc	0.954	0.000	−0.240	2.074
Ti	0.796	0.564	−0.210	1.985
V	0.604	1.851	−0.192	1.951
Cr	0.632	3.290	−0.217	1.957
Mn	0.525	3.576	−0.189	1.907
Fe	0.414	2.440	−0.172	1.897
Co	0.273	1.219	−0.162	1.884
Ni	0.241	0.000	−0.158	1.896
Cu	0.475	0.506	−0.214	1.960
Zn	0.580	0.000	−0.2187	2.060

**Table 2:** Identified catalytic rate-determining step for CO_2_ electrocatalysis using TM–HHTQ, with limiting potential ($U_{L}$/V) and overpotential (η/V).

TM–HHTQ	Rate-Determining Step	$U_{L}$	Product	η
Sc	* + CO_2_ + H^+^ + e^−^ → *COOH	−1.173	CO	1.067
			HCHO	1.103
			CH_4_	1.342
Ti	*OCHOH → * + HCOOH	−0.856	HCOOH	0.606
V	*CO + H_2_O + H^+^ + e^−^ → *CHO + H_2_O	−0.506	CH_4_	0.675
Cr	*OCHOH → * + HCOOH	−0.486	HCOOH	0.236
Mn	* + CO_2_ + H^+^ + e^−^ → *COOH	−0.611	CO	0.505
			HCHO	0.541
			CH_3_OH	0.627
			CH_4_	0.78
Fe	* + CO_2_ + H^+^ + e^−^ →*COOH	−0.412	HCHO	0.342
Co	* + CO_2_ + H^+^ + e^−^ → *COOH	−0.572	CO	0.428
			HCHO	0.466
			CH_3_OH	0.502
			CH_4_	0.588
Ni	* + CO_2_ + H^+^ + e^−^ → *COOH	−0.727	CO	0.741
			HCHO	0.621
			CH_3_OH	0.657
			CH_4_	0.743
Cu	* + CO_2_ + H^+^ + e^−^ → *OCHO	−1.030	CH_3_OH	0.96
Zn	* + CO_2_ + H^+^ + e^−^ →*COOH	−0.860	CO	1.046

## Data Availability

The data presented in this study are available in article and Supplementary Materials.

## Supplementary Materials

The following supporting information can be downloaded at: https://www.mdpi.com/article/10.3390/molecules29122896/s1, Table S1. $E_{c}$ is the cohesive energy of the bulk TM, $E_{b}$ is the binding energy between the TM and the TM–HHTQ, where TM are the metal atoms of the first transition metal series. Table S2. Gibbs free energy change (ΔG/eV) of the first protonation step in the CO_2_ reduction reaction (CO_2_RR) and H_2_ evolution reaction (HER) on the TM–HHTQ. Table S3. Adsorption Energy ($E_{ads}$/eV) of different CO_2_ reduction products. Table S4–S13. Gibbs free energy change for each protonation step of TM-HHTQ electrocatalytic CO_2_ reduction.
