# Supplementary material for: Exploring the CO2 Electrocatalysis Potential of 2D Metal–Organic Transition Metal–Hexahydroxytriquinoline Frameworks: A DFT Investigation
Source: Molecules. 2024 Jun 18;29(12):2896. doi: 10.3390/molecules29122896 (PMC11206698; doi:10.3390/molecules29122896)
Supplement: Supplementary file 1 [file molecules-29-02896-s001.zip › molecules-3044667-supplementary.pdf]

Exploring the CO_2 Electrocatalysis Potential of 2D Metal–Organic Transition Metal–Hexahydroxytriquinoline Frameworks: A DFT Investigation

Yufeng Wen[1], Daguo Jiang[1], Zhangli Lai[1]* , Xianshi Zeng[1]*, Bo Liu[1], Yanan Xiao[1], Wen Ruan[1], and Kai Xiong[2,3]

1 School of Mathematical Sciences and Physics, Jinggangshan University, Ji'an 343009, China; jgsuwyf@sina.com(Y.W.);jgsujdg1968@163.com(D.J.); liubo@jgsu.edu.cn(B.L.);yananxiao06@163.com(Y.X.);ruanwen@jgsu.edu.cn(W.R.)

2 Materials Genome Institute, National Center for International Research on Photoelectric and Energy Materials, School of Materials and Energy, Yunnan University, Kunming 650091, China; xiongkai@ynu.edu.cn

3 Advanced Computing Center, Information Technology Center, Yunnan University, Kunming 650091, China.

* Correspondence:;15979627768@163.com(Z.L.);zengxueliang@163.com(X.Z.).

Table S 1 E_c is the cohesive energy of the bulk TM, E_b is the binding energy between the TM and the TM–HHTQ, where TM are the metal atoms of the first transition metal series.

TM-HHTQ	E_b(eV)	E_c(eV)
Sc	-12.494	-4.422
Ti	-12.404	-5.955
V	-10.765	-6.577
Cr	-8.823	-4.010
Mn	-9.353	-3.760
Fe	-8.990	-5.263
Co	-8.514	-6.050
Ni	-8.002	-6.016
Cu	-5.853	-3.430
Zn	-4.475	-1.055

Table S 2 Gibbs free energy change (ΔG/eV) of the first protonation step in the CO_2 reduction reaction (CO_2RR) and H_2 evolution reaction (HER) on the TM–HHTQ

TM-HHTQ	**ΔG[*H]**	**ΔG*COOH**	**ΔG*OCHO**
Sc	1.352	1.173	-0.064
Ti	0.201	0.209	-0.614
V	0.278	0.449	0.018
Cr	0.449	0.439	0.290
Mn	0.553	0.611	0.802
Fe	0.432	0.412	1.072
Co	0.633	0.572	1.356
Ni	0.833	0.727	1.378
Cu	1.634	1.219	1.030
Zn	1.174	0.860	1.648

Table S 3 Adsorption Energy (Eads/eV) of different CO_2 reduction products

TM-HHTQ	CO	HCOOH	HCHO	CH_3OH	CH_4
Sc	-0.609	-1.027	-0.940	-1.178	-0.224
Ti	-0.999	-0.759	-1.209	-1.444	-0.238
V	-1.06	-1.062	-1.068	-1.200	-0.099
Cr	-0.243	-0.746	-0.805	-0.899	-0.183
Mn	-0.361	-0.525	-0.530	-0.667	-0.131
Fe	-0.219	-0.568	-0.581	-0.714	-0.061
Co	-0.167	-0.147	-0.174	-0.072	-0.056
Ni	-0.172	-0.191	-0.230	-0.304	-0.090
Cu	-0.252	-1.041	-0.196	-0.392	-0.154
Zn	-0.047	-0.208	-1.258	-1.376	-0.886

Table S 4 Gibbs free energy change for each protonation step of Zn-HHTQ electrocatalytic CO_2 reduction.

$n(H^+ + e^-)$transferred	Chemical reaction equation	ΔG
1	$* + CO_2 + H^+ + e^- \rightarrow *COOH$	0.860
	$* + CO_2 + H^+ + e^- \rightarrow *OCHO$	1.648
2	$*COOH + H^+ + e^- \rightarrow *CO + H_2O$	-0.984
3	$*CO + H_2O + H^+ + e^- \rightarrow *CHO + H_2O$	1.095
	$*CO + H_2O + H^+ + e^- \rightarrow *COH + H_2O$	1.802
	$*CO + H_2O \rightarrow * + CO + H_2O$	0.819

Table S 5 Gibbs free energy change for each protonation step of Ti-HHTQ electrocatalytic CO_2 reduction.

$n(H^+ + e^-)$transferred	Chemical reaction equation	ΔG
1	$* + CO_2 + H^+ + e- \rightarrow *COOH$	0.210
	$* + CO_2 + H^+ + e^- \rightarrow *OCHO$	-0.614
2	$*COOH + H^+ + e^- \rightarrow *CO + H_2O$	-0.595
	$*OCHO + H^+ + e^- \rightarrow *OCHOH$	-0.422
3	$*CO + H_2O + H^+ + e^- \rightarrow *CHO + H_2O$	0.986
	$*CO + H_2O + H^+ + e^- \rightarrow *COH + H_2O$	2.007
	$*OCHOH + H^+ + e^- \rightarrow *CHO + H_2O$	1.637
	$*OCHOH + H^+ + e^- \rightarrow *OCH + H_2O$	2.092
	$*OCHOH \rightarrow *+HCOOH$	0.856

Table S 6 Gibbs free energy change for each protonation step of Cr-HHTQ electrocatalytic CO_2 reduction.

$n(H^+ + e^-)$transferred	Chemical reaction equation	ΔG
1	$* + CO_2 + H^+ + e^- \rightarrow *COOH$	0.439
	$* + CO_2 + H^+ + e^- \rightarrow *OCHO$	0.290
2	$*COOH + H^+ + e^- \rightarrow *CO + H_2O$	-0.701
	$*OCHO + H^+ + e^- \rightarrow *OCHOH$	-0.957
3	$*CO + H_2O + H^+ + e^- \rightarrow *CHO + H_2O$	0.582
	$*CO + H_2O + H^+ + e^- \rightarrow *COH + H_2O$	2.121
	$*OCHOH + H^+ + e^- \rightarrow *CHO + H_2O$	0.988
	$*OCHOH + H^+ + e^- \rightarrow *OCH + H_2O$	1.993
	$*CO + H_2O \rightarrow * + CO + H_2O$	1.068
	$*OCHOH \rightarrow * + HCOOH$	0.486

Table S 7 Gibbs free energy change for each protonation step of V-HHTQ electrocatalytic CO_2 reduction.

n(H^++e^-)transferred	Chemical reaction equation	ΔG
1	$* + CO_2 + H^+ + e^- \rightarrow *COOH$	0.449
	$* + CO_2 + H^+ + e^- \rightarrow *OCHO$	0.018
2	$*COOH + H^+ + e^- \rightarrow *CO + H_2O$	-0.541
	$*OCHO + H^+ + e^- \rightarrow *OCHOH$	-0.647
3	$*CO + H_2O + H^+ + e^- \rightarrow *CHO + H_2O$	0.506
	$*CO + H_2O + H^+ + e^- \rightarrow *COH + H_2O$	1.504
	$*OCHOH + H^+ + e^- \rightarrow *CHO + H_2O$	1.042
	$*OCHOH + H^+ + e^- \rightarrow *OCH + H_2O$	2.064
4	$*CHO + H_2O + H^+ + e^- \rightarrow *OCH_2 + H_2O$	-0.945
5	$*OCH_2 + H_2O + H^+ + e^- \rightarrow *OCH_3 + H_2O$	-0.602
6	$*OCH_3 + H_2O + H^+ + e^- \rightarrow *CH_3OH + H_2O$	-0.465
	$*OCH_3 + H_2O + H^+ + e^- \rightarrow *O + CH_4 + H_2O$	-0.410
7	$*O + CH_4 + H_2O + H^+ + e^- \rightarrow *OH + CH_4 + H_2O$	-0.783
	$*CH_3OH + H_2O + H^+ + e^- \rightarrow *OH + CH_4 + H_2O$	-0.728
8	$*OH + CH_4 + H_2O + H^+ + e^- \rightarrow * + CH_4 + 2H_2O$	0.245

Table S 8 Gibbs free energy change for each protonation step of Cu-HHTQ electrocatalytic CO_2 reduction.

n(H^++e^-)transferred	Chemical reaction equation	ΔG
1	$* + CO_2 + H^+ + e^- \rightarrow *COOH$	1.219
	$* + CO_2 + H^+ + e^- \rightarrow *OCHO$	1.030
2	$*OCHO + H^+ + e^- \rightarrow *OCHOH$	-1.247
3	$*OCHOH + H^+ + e^- \rightarrow *CHO + H_2O$	0.829
	$*OCHOH + H^+ + e^- \rightarrow *OCH + H_2O$	1.255
4	$*CHO + H_2O + H^+ + e^- \rightarrow *OCH_2 + H_2O$	-1.554
5	$*OCH_2 + H_2O + H^+ + e^- \rightarrow *OCH_3 + H_2O$	0.662
	$*OCH_2 + H_2O + H^+ + e^- \rightarrow * + HCHO + H_2O + H^+ + e^-$	0.960
6	$*OCH_3 + H_2O + H^+ + e^- \rightarrow *CH_3OH + H_2O$	-0.962
	$*OCH_3 + H_2O + H^+ + e^- \rightarrow *O + CH_4 + H_2O$	2.610
7	$*CH_3OH + H_2O + H^+ + e^- \rightarrow *OH + CH_4 + H_2O$	1.125
	$*CH_3OH + H_2O \rightarrow * + CH_3OH + H_2O$	0.257

Table S 9 Gibbs free energy change for each protonation step of Fe-HHTQ electrocatalytic CO_2 reduction.

$n(H^+ + e^-)$ transferred	Chemical reaction equation	ΔG
1	$* + CO_2 + H^+ + e^- \rightarrow *COOH$	0.412
	$* + CO_2 + H^+ + e^- \rightarrow *OCHO$	1.072
2	$*COOH + H^+ + e^- \rightarrow *CO + H_2O$	0.084
3	$*CO + H_2O + H^+ + e^- \rightarrow *CHO + H_2O$	-0.198
	$*CO + H_2O + H^+ + e^- \rightarrow *COH + H_2O$	1.478
	$*CO + H_2O \rightarrow * + CO + H_2O$	0.565
4	$*CHO + H_2O + H^+ + e^- \rightarrow *OCH_2 + H_2O$	-0.333
5	$*OCH_2 + H_2O + H^+ + e^- \rightarrow *OCH_3 + H_2O$	0.238
	$*OCH_2 + H_2O + H^+ + e^- \rightarrow * + HCHO + H_2O + H^+ + e^-$	0.388
6	$*OCH_3 + H_2O + H^+ + e^- \rightarrow *CH_3OH + H_2O$	-1.337
	$*OCH_3 + H_2O + H^+ + e^- \rightarrow *O + CH_4 + H_2O$	0.533
7	$*CH_3OH + H_2O + H^+ + e^- \rightarrow *OH + CH_4 + H_2O$	0.475
	$*CH_3OH + H_2O \rightarrow * + CH_3OH + H_2O$	0.337
8	$*OH + CH_4 + H_2O + H^+ + e^- \rightarrow * + CH_4 + 2H_2O$	-1.216

Table S 10 Gibbs free energy change for each protonation step of Co-HHTQ electrocatalytic CO_2 reduction.

n(H^++e^-)transferred	Chemical reaction equation	ΔG
1	$* + CO_2 + H^+ + e^- \rightarrow *COOH$	0.572
	$* + CO_2 + H^+ + e^- \rightarrow *OCHO$	1.356
2	$*COOH + H^+ + e^- \rightarrow *CO + H_2O$	-0.182
3	$*CO + H_2O + H^+ + e^- \rightarrow *CHO + H_2O$	-0.369
	$*CO + H_2O + H^+ + e^- \rightarrow *COH + H_2O$	1.378
	$*CO + H_2O \rightarrow * + CO + H_2O$	0.241
4	$*CHO + H_2O + H^+ + e^- \rightarrow *OCH_2 + H_2O$	-0.048
5	$*OCH_2 + H_2O + H^+ + e^- \rightarrow *OCH_3 + H_2O$	0.450
	$*OCH_2 + H_2O + H^+ + e^- \rightarrow * + HCHO + H_2O + H^+ + e^-$	-0.140
6	$*OCH_3 + H_2O + H^+ + e^- \rightarrow *CH_3OH + H_2O$	-1.493
	$*OCH_3 + H_2O + H^+ + e^- \rightarrow *O + CH_4 + H_2O$	0.798
7	$*CH_3OH + H_2O + H^+ + e^- \rightarrow *OH + CH_4 + H_2O$	0.289
	$*CH_3OH + H_2O \rightarrow * + CH_3OH + H_2O$	0.254
8	$*OH + CH_4 + H_2O + H^+ + e^- \rightarrow * + CH_4 + 2H_2O$	-1.841

Table S 11 Gibbs free energy change for each protonation step of Mn-HHTQ electrocatalytic CO_2 reduction.

$n(H^{+}+e^{-})$transferred	Chemical reaction equation	ΔG
1	$* + CO_2 + H^+ + e^- \rightarrow *COOH$	0.611
	$* + CO_2 + H^+ + e^- \rightarrow *OCHO$	0.802
2	$*COOH + H^+ + e^- \rightarrow *CO + H_2O$	-0.420
3	$*CO + H_2O + H^+ + e^- \rightarrow *CHO + H_2O$	0.004
	$*CO + H_2O + H^+ + e^- \rightarrow *COH + H_2O$	1.787
	$*CO + H_2O \rightarrow * + CO + H_2O$	0.377
4	$*CHO + H_2O + H^+ + e^- \rightarrow *OCH_2 + H_2O$	-0.606
5	$*OCH_2 + H_2O + H^+ + e^- \rightarrow *OCH_3 + H_2O$	0.163
	$*OCH_2 + H_2O + H^+ + e^- \rightarrow * + HCHO + H_2O + H^+ + e^-$	0.197
6	$*OCH_3 + H_2O + H^+ + e^- \rightarrow *CH_3OH + H_2O$	-1.290
	$*OCH_3 + H_2O + H^+ + e^- \rightarrow *O + CH_4 + H_2O$	0.591
7	$*O + CH_4 + H_2O + H^+ + e^- \rightarrow *OH + CH_4 + H_2O$	-1.695
	$*CH_3OH + H_2O + H^+ + e^- \rightarrow *OH + CH_4 + H_2O$	0.186
	$*CH_3OH + H_2O \rightarrow * + CH_3OH + H_2O$	0.346
8	$*OH + CH_4 + H_2O + H^+ + e^- \rightarrow * + CH_4 + 2H_2O$	-1.130

Table S 12 Gibbs free energy change for each protonation step of Ni-HHTQ electrocatalytic CO_2 reduction.

$n(H^{+}+e^{-})$transferred	Chemical reaction equation	ΔG
1	$* + CO_2 + H^+ + e^- \rightarrow *COOH$	0.726
	$* + CO_2 + H^+ + e^- \rightarrow *OCHO$	1.378
2	$*COOH + H^+ + e^- \rightarrow *CO + H_2O$	-0.386
3	$*CO + H_2O + H^+ + e^- \rightarrow *CHO + H_2O$	-0.221
	$*CO + H_2O + H^+ + e^- \rightarrow *COH + H_2O$	1.419
	$*CO + H_2O \rightarrow * + CO + H_2O$	0.324
4	$*CHO + H_2O + H^+ + e^- \rightarrow *OCH_2 + H_2O$	-0.231
5	$*OCH_2 + H_2O + H^+ + e^- \rightarrow *OCH_3 + H_2O$	0.676
	$*OCH_2 + H_2O + H^+ + e^- \rightarrow * + HCHO + H_2O + H^+ + e^-$	0.087
6	$*OCH_3 + H_2O + H^+ + e^- \rightarrow *CH_3OH + H_2O$	-1.709
	$*OCH_3 + H_2O + H^+ + e^- \rightarrow *O + CH_4 + H_2O$	0.416
7	$*O + CH_4 + H_2O + H^+ + e^- \rightarrow *OH + CH_4 + H_2O$	-1.513
	$*CH_3OH + H_2O + H^+ + e^- \rightarrow *OH + CH_4 + H_2O$	0.612
	$*CH_3OH + H_2O \rightarrow * + CH_3OH + H_2O$	0.030
8	$*OH + CH_4 + H_2O + H^+ + e^- \rightarrow * + CH_4 + 2H_2O$	-2.086

Table S 13 Gibbs free energy change for each protonation step of Sc-HHTQ electrocatalytic CO_2 reduction.

$n(H^+ + e^-)$transferred	Chemical reaction equation	ΔG
1	$* + CO_2 + H^+ + e^- \rightarrow *COOH$	1.173
	$* + CO_2 + H^+ + e^- \rightarrow *OCHO$	-0.064
2	$*COOH + H^+ + e^- \rightarrow *CO + H_2O$	-1.130
	$*OCHO + H^+ + e^- \rightarrow *OCHOH$	-0.834
3	$*CO + H_2O + H^+ + e^- \rightarrow *CHO + H_2O$	0.924
	$*CO + H_2O + H^+ + e^- \rightarrow *COH + H_2O$	2.273
	$*OCHOH + H^+ + e^- \rightarrow *CHO + H_2O$	1.865
	$*OCHOH + H^+ + e^- \rightarrow *OCH + H_2O$	2.031
	$*CO + H_2O \rightarrow * + CO + H_2O$	0.671
4	$*CHO + H_2O + H^+ + e^- \rightarrow *OCH_2 + H_2O$	-1.734
5	$*OCH_2 + H_2O + H^+ + e^- \rightarrow *OCH_3 + H_2O$	-0.051
	$*OCH_2 + H_2O + H^+ + e^- \rightarrow * + HCHO + H_2O + H^+ + e^-$	0.994
6	$*OCH_3 + H_2O + H^+ + e^- \rightarrow *CH_3OH + H_2O$	-1.127
	$*OCH_3 + H_2O + H^+ + e^- \rightarrow *O + CH_4 + H_2O$	1.488
7	$*CH_3OH + H_2O + H^+ + e^- \rightarrow *OH + CH_4 + H_2O$	0.083
8	$*OH + CH_4 + H_2O + H^+ + e^- \rightarrow * + CH_4 + 2H_2O$	-0.366
